# Sellar Metastasis of Cervical Adenocarcinoma

**DOI:** 10.1155/2019/9769657

**Published:** 2019-01-10

**Authors:** Krishnan Ravindran, Brandon M. Zsigray, Michael P. Wemhoff, John D. Spencer, Ewa Borys, Chirag R. Patel, Anand V. Germanwala

**Affiliations:** ^1^Department of Neurological Surgery, Loyola University Medical Center, Maywood, Illinois, USA; ^2^Department of Pathology, Loyola University Medical Center, Maywood, Illinois, USA; ^3^Department of Otolaryngology Stritch School of Medicine, Loyola University Medical Center, Maywood, Illinois, USA

## Abstract

**Background:**

Pituitary metastasis of cervical adenocarcinoma is an exceedingly rare phenomenon.

**Case Description:**

The authors present a case of a 66-year-old female with cervical adenocarcinoma who was discovered to have a rapidly growing intrasellar mass in the work-up of adrenal insufficiency and hypothyroidism. The patient underwent subsequent endoscopic endonasal subtotal resection of the mass. Histopathological analysis of the resected lesion demonstrated features consistent with metastatic mucinous adenocarcinoma of the cervix. While initially neurologically asymptomatic following surgery, the patient developed an oculomotor nerve palsy several weeks following surgical debulking, at which time neuroimaging revealed marked regrowth and suprasellar extension of the metastatic lesion.

**Conclusions:**

While metastatic cervical adenocarcinoma to the sella is rare, it should be considered in the differential based on the history of the patient.

## 1. Introduction

Metastatic lesions to the sella are believed to comprise only 1% of all pituitary lesions, of which the most common sites of primary tumor origin are the breast and lung [1–3]. Compression of parasellar anatomical structures may produce symptoms such as visual field loss, headache and ophthalmoplegia [4]. Cerebral metastasis of cervical malignancy is uncommon [5]. To our knowledge, only one other case of cervical carcinoma with pituitary metastasis has been reported in a nonautopsy setting [6]. Here, we report a case of sellar metastasis in a patient with cervical adenocarcinoma that was debulked endoscopically, highlight the clinical findings and natural history of this rare presentation, and review the literature.

## 2. Case Presentation

A 66-year-old female with a history of mucinous adenocarcinoma of the cervix presented to the neurosurgery outpatient clinic for evaluation of a sellar mass found during workup of adrenal insufficiency and hypothyroidism. The patient did not have unusual headaches or vision problems. Three months prior to discovery of the sellar mass, she was diagnosed with stage IIb mucinous adenocarcinoma of the cervix and was treated with chemotherapy. At the time of neurosurgery clinic presentation, she was neurologically intact, including full visual fields. Laboratory work-up demonstrated pituitary insufficiency with central hypothyroidism.

**Figure 1:**
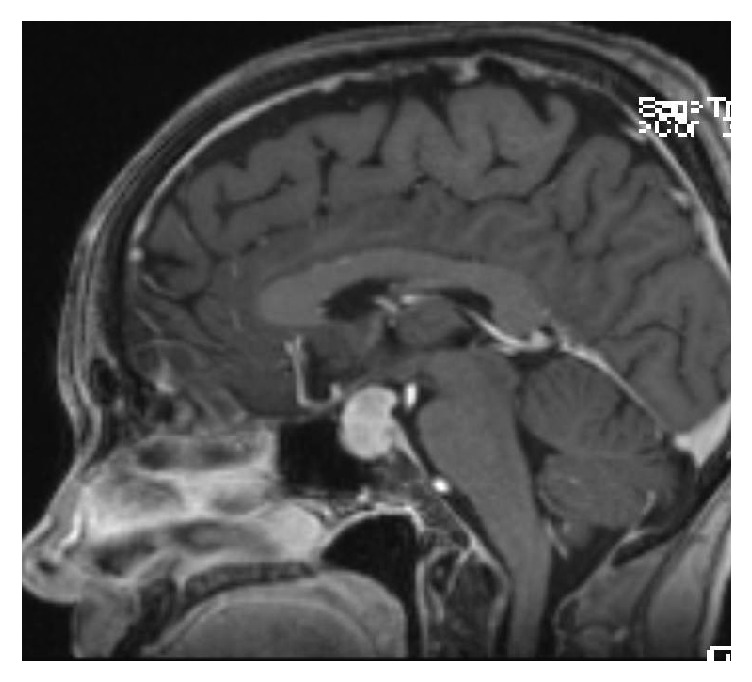
Preoperative sagittal MRI with contrast demonstrating the homogenously contrast enhancing sellar/suprasellar mass with mass effect on the optic chiasm.

The initial magnetic resonance imaging (MRI) revealed a 1.8 × 1.1 cm contrast-enhancing mass within the sella, with extension to the suprasellar cistern and optic chiasm abutment. Preoperative imaging obtained the following month in preparation for surgery demonstrated that the mass had grown to 2.2 × 1.5 cm (Figure 1).

**Figure 2:**
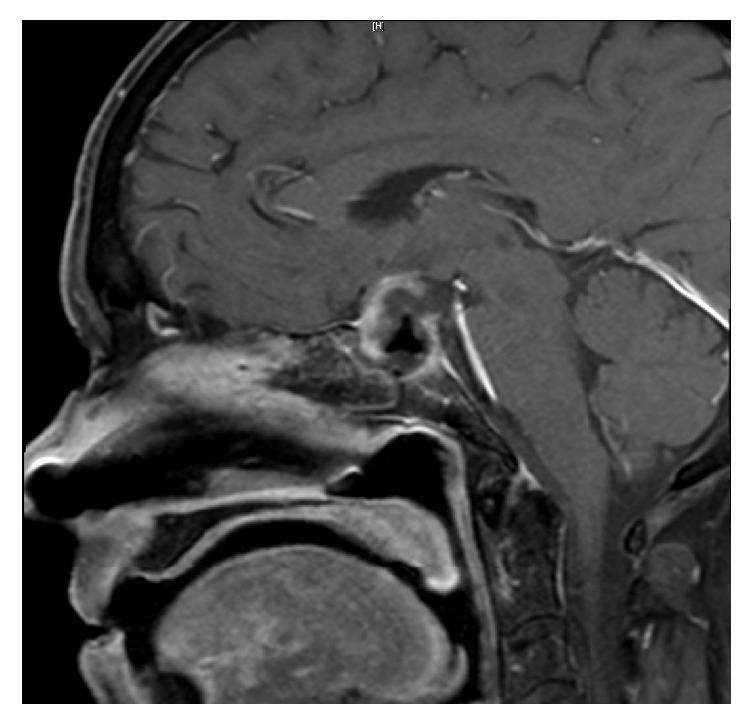
Immediately postoperative sagittal MRI with contrast showing subtotal resection through an endoscopic endonasal approach.

**Figure 3:**
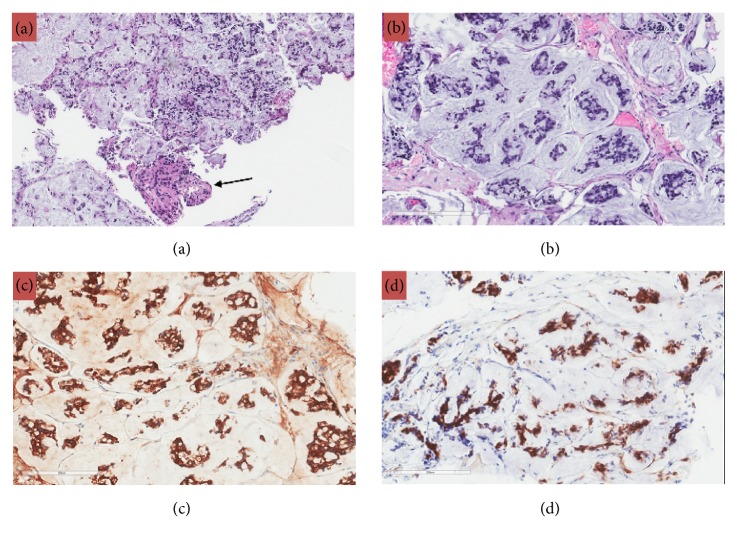
(a) Frozen section tissue showing interface between mucinous adenocarcinoma metastasis and adjacent adenohypophysis (arrow), H&E, 40x. (b) Metastatic mucinous adenocarcinoma showing clusters of moderately pleomorphic epithelial cells floating in pools of mucin separated by fibrovascular septae of variable thickness, H&E, 200x. (c) Cytokeratin CK8/18 is positive in tumor cells; immunohistochemical stain, 200x. (d) p16 shows nuclear positivity; immunohistochemical stain, 200x.

**Figure 4:**
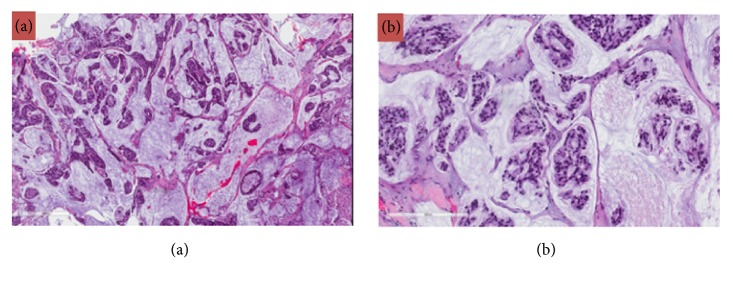
(a, b) Cervical biopsy showing a moderately differentiated mucinous/colloid adenocarcinoma. H&E, 40x (a) and 200x (b).

The patient underwent an endoscopic endonasal approach for resection of the intradural sellar mass. Intraoperative findings demonstrated a very firm, infiltrative, vascular mass with dense adherence to surrounding structures, including the dura, medical cavernous walls, and diaphragma. Intraoperative frozen section pathology was consistent with metastatic carcinoma. The tumor was debulked until normal appearing pituitary tissue was identified and the margin of tumor adherence to the diaphragma was reached. Postoperatively, the patient did well without new hormonal deficiencies or vision problems. A subtotal resection (>80%) was achieved (Figure 2). Gross histology and immunohistochemical staining ultimately confirmed the diagnosis of metastatic mucinous adenocarcinoma of the cervix (Figures 3 and 4).

**Figure 5:**
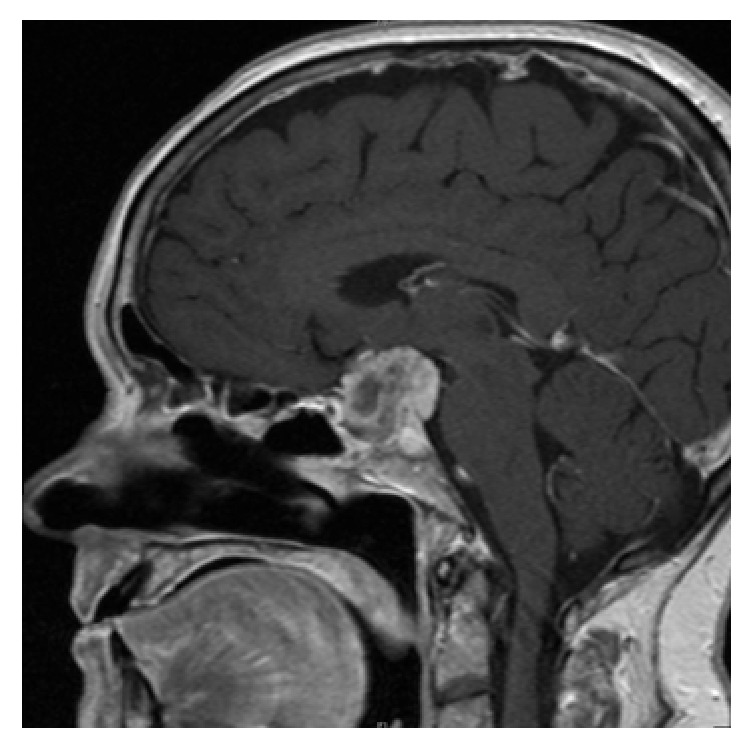
Postoperative sagittal MRI with contrast five weeks after surgery showing significant tumor recurrence.

The patient's immediate postoperative course was unremarkable. Given the diagnosis and intraoperative/postoperative findings of subtotal resection, adjuvant chemoradiation therapy was encouraged but the patient refused additional treatment. She was discharged home two days after surgery. She developed decreased left eye visual acuity and ptosis one week after surgery. A CT of the head at this time did not show any intracranial hemorrhage and a repeat MRI showed new enhancement suggestive of tumor recurrence within the sellar and suprasellar regions. The patient was started on steroids but declined any further treatment, including repeat surgery. Her ophthalmic symptoms ultimately progressed to a complete left cranial nerve III palsy four weeks after surgery. Follow-up MR imaging at five weeks after surgery revealed significant progression of the tumor to 2.9 × 2.4 cm with significant suprasellar extension (Figure 5). Though a computed tomography scan of the chest, abdomen, and pelvis at this time demonstrated no new neoplastic burden, a radiotracer bone scan demonstrated likely new metastatic lesions in the skull, bilateral humeri, bilateral acetabula, bilateral femurs, and the lumbosacral vertebrae. After further discussion with her gynecologic oncologist and radiation oncology, the patient again refused pursuing any further treatment, including palliative radiation or systemic therapies and elected to pursue home hospice. The patient died approximately two months after surgery.

## 3. Discussion

Metastases to the sellar region have been hypothesized to occur either hematogenously, such as from the pituitary stalk or cavernous sinus, or via leptomeningeal spread [7]. Pituitary metastasis typically occurs late in disease course, carrying a poor prognosis [8]. Metastases appear to display a predilection for the posterior lobe, likely owing to greater vascularity of the posterior lobe from the hypophyseal arteries, as compared to the anterior lobe, which is supplied by the portal system [6, 8]. Indeed, it has been suggested that metastatic involvement of the anterior lobe is in fact due to contiguous extension from the posterior lobe [9].

Pituitary adenomas and metastatic lesions to the parasellar region are nearly indistinguishable on neuroimaging. On plain film X-ray images, sellar enlargement or deformity and erosion of the sellar floor may be seen in both metastatic and benign lesions [10]. On MRI, contrast enhancement has been reported to be a prominent feature more commonly observed in metastatic lesions [10]. Metastatic lesions may also exhibit a “dumbbell” appearance due to indentation at the level of the diaphragm [11]. In this case, extension to the suprasellar cistern was seen with “minimal” bony invasion, also previously described as a predominant feature of metastatic lesions [10, 11]. Interestingly, despite radiological evidence of optic chiasm displacement on preoperative MRI, the patient's visual fields remained intact, though the patient progressed to suffer poor visual acuity and a left oculomotor nerve palsy following tumor debulking over a few weeks. It has been suggested that oculomotor nerve dysfunction may be a distinguishing clinical feature between metastatic and primary lesions given that compression of the intracavernous segment of this nerve by pituitary adenomas is rare [11].

Cervical carcinoma typically disseminates locally via the lymphatics to the retroperitoneal lymph nodes; distant hematogenous metastasis most commonly occurs to the liver, lung, and bone [12]. Cerebral metastasis is rare with an estimated prevalence of 0.5-1.0% and portends a particularly poor prognosis [5]. One study found that in postmortem examination of 60 patients with sellar lesions, 3 cases of primary cervical carcinoma were observed, none of which demonstrated sellar metastasis [2]. To our knowledge, only one other case of cervical carcinoma metastasis to the pituitary has been reported [6]. In a 2000 study, Salpietro and colleagues described a sellar/suprasellar metastasis of an epidermoid cervical carcinoma in a 44-year-old female who presented with diabetes insipidus and diplopia. Radiation therapy of the primary cervical lesion had been done two years prior to presentation. Despite transsphenoidal debulking, the patient's condition deteriorated in the context of widespread metastatic disease to the lung and bone, and the patient died within several months.

For brain metastasis from cervical cancer, combination therapy of either chemotherapy or stereotactic radiosurgery followed by whole-brain irradiation has been shown to extend median survival to up to 4.6 months [13, 14]. The majority of these studied cases have been squamous cell carcinomas with an intraparenchymal location. Additionally, the majority of these lesions have been histologically poorly differentiated with high tumor grade [15]. Multimodality therapies are generally recommended with adjuvant chemoradiation therapy following maximal safe surgical resection. This case highlights the importance of initiating multimodality therapy expeditiously.

## 4. Conclusion

While cerebral metastases of cervical malignancies have been described, metastasis of well-differentiated cervical malignancy to the pituitary region is rare. However, this should be considered in the differential diagnosis of patients with this history and based on the clinical presentation. The development of rapid endocrinopathies, oculomotor dysfunction, and quick radiographic progression suggest a more aggressive neoplasm. Maximally safe resection should be performed, including decompression of the optic apparatus, followed by efficient consideration for adjuvant therapy.
